# Social Capital, Environmental Knowledge, and Pro-Environmental Behavior

**DOI:** 10.3390/ijerph19031443

**Published:** 2022-01-27

**Authors:** Qinyuan Wan, Wencui Du

**Affiliations:** School of Economics, Capital University of Economics and Business, Beijing 100070, China; wanqy001@163.com

**Keywords:** social capital, public domain environmental behavior, private domain environmental behavior

## Abstract

As the value form of public access to environmental information, the impact of social capital on pro-environmental behavior cannot be ignored. Based on the data of the Chinese General Social Survey 2013 (CGSS2013), this study measures social capital from four aspects—social trust, social norms, social network, and social participation—and it empirically tests the impact of social capital on private and public pro-environmental behavior. The study finds that social capital helps promote pro-environmental behavior. Specifically, the more the public abides by social norms, the higher the degree of social participation, and the stronger the willingness to adopt private and public pro-environmental behaviors. However, the improvement of social trust only has a significant impact on the private environmental behaviors, and the expansion of the social network scale only has a significant impact on the public pro-environmental behaviors. The enhancement of social capital enriches environmental knowledge and promotes pro-environmental behaviors. The mechanism test shows that environmental knowledge plays an intermediary role in the path of social capital affecting individual pro-environmental behavior. The improvement of social capital has a significant impact on the environmental knowledge of individuals with high subjective social class. The gender heterogeneity of social capital affecting environmental knowledge mainly stems from social trust and social network. The stronger the degree of social trust, the richer the environmental knowledge of women, and the social network mainly affects the knowledge level of men. In addition, the publics in the southern region are more likely to be affected by social trust and improve environmental knowledge. Based on the above research conclusions, this paper puts forward policy suggestions on institutional aspects, such as increasing support for informal environmental organizations, carrying out differentiated sustainable development education, and improving the mechanism of environmental information communication.

## 1. Introduction

To further promote the modernization of the ecological environmental governance system and capacity, in March 2020, the General Office of the Central Committee of the Communist Party of China and the General Office of the State Council issued the Guidance on Building a Modern Environmental Governance System, which clearly stated that the participation channels of the public in environmental governance should be unimpeded, and a good pattern of social participation in environmental governance should be further formed. Public participation in environmental protection will largely reduce the overall cost of environmental governance and improve governance efficiency. Therefore, it is particularly important to promote the public to take pro-environmental behavior and improve environmental participation.

Promoting the formation of public pro-environmental behavior and public participation in environmental governance has not been proposed in recent years but rather gradually formed with the development of China’s environmental protection cause. In 1989, the 11th session of the Standing Committee of the 17th National People’s Congress passed the Environmental Protection Law of the People’s Republic of China. The general part of the environmental protection law points out that “all the units and individuals have the obligation to protect the environment”. However, the law does not specify how the public should fulfill its obligation to protect the environment. Until 2020, the Guidance on Building a Modern Environmental Governance System pointed out that we should “adhere to the party’s centralized and unified leadership as the leadership, to strengthen the leading role of the government as the key, to deepen the main role of enterprises as the fundamental, to better mobilize social organizations and public participation as the support”. In the process of ecological environment governance in China, the public has experienced a conceptual change from understanding to participation, and it gradually formed a consensus on pro-environmental behavior, forming a multidimensional pro-environmental behavior system.

To achieve the construction of ecological civilization, it is necessary to change the way of production and life, and pro-environmental behavior plays an important role. From the perspective of production, the public is the final consumer of the product, and the public’s environmental awareness and behavior will be directly reflected in the consumer choice, which forces the formation of a green production mode. From the perspective of life, as the main body of life, environmental awareness and behavior will directly affect the formation of a green lifestyle. Public participation in environmental protection can overcome the shortcomings of market and government regulation and play an irreplaceable role in many ways [1]. However, under the existing environmental governance system in China, the public’s environmental awareness is slightly insufficient, resulting in the inherent obstacles of public participation in environmental protection. At the same time, the government’s environmental regulation is mainly aimed at enterprises, lacking the encouragement and regulation of pro-environmental behavior, resulting in external obstacles for the public to participate in environmental protection.

Therefore, it is important to clarify the factors affecting public pro-environmental behavior and find ways to promote pro-environmental behavior. Existing studies attribute the influencing factors of pro-environmental behavior to cognitive factors, demographic factors, social structure factors and cultural factors. Cognitive factors [2,3,4] mainly focus on the one-way output mode of the public for the environment, from rational environmental knowledge to personal sensory experience; these kinds of factors focus on the individual’s environmental endowments and the individual–environment path to analyze their pro-environmental behavior. Demographic factors, as the name suggests, mainly include the social characteristics of individuals, such as gender, income, and other aspects [5,6,7,8] to study the impact of social endowments on public environmental tendencies. Compared with cognitive factors, demographic factors can be seen as external factors at the individual level.

At the social level, the research on social hierarchy and social differentiation forms [9,10] reveals the influence of the changes of social environment on human beings’ environmental selection behavior. Social structure factors are rooted in the individual’s survival mode, that is, nature and society, and explain the unity of opposites of the two. Cultural factors [11,12,13] are complementary to social structure factors. Culture was born in the combination and development of nature and society. Social structure factors separate the relationship between nature and society, and they study individuals’ natural behavior from the social level alone, while cultural factors highlight the combined effect of nature and society on pro-environmental behavior.

The existing literature studies the pro-environmental behavior of the public from the internal and external mechanisms of the individual–environment (cognitive factors and demographic factors) or the separation and integration of society and nature (social structure factors and cultural factors). However, these two types of research paths are difficult to reach a joint explanation between individuals, society, and nature. There is a lack of detailed explanation and interpretation on the individual–society path, and the impact of social capital on individual pro-environmental behavior is ignored. Therefore, this paper hopes to introduce the influencing factor of social capital, aiming at the connection between individuals and society, and indirectly improve public pro-environmental behavior. Social capital refers to the social network, trust, and norms that can improve economic efficiency through coordinated actions [14], including social trust, social norms, social network, and social participation [15]. It is a kind of value concept that is subjectively shared. It can enable the public to reach spiritual or attitude cooperation and consensus, thereby promoting pro-environmental behavior. This study measures individual social capital from four aspects of social trust, social norms, social networks, and social participation, and it measures people’s pro-environmental behavior from two dimensions of the private domain and public domain. Using the microdata of the China General Social Survey (CGSS) in 2013, based on ordinary least squares (OLS) regression, this study examines the impact of social capital on pro-environmental behavior in the private domain and public domain.

Compared with the existing literature, the marginal contribution of this paper is reflected in the first systematic discussion of the impact of social capital on pro-environmental behavior, and it supplements the existing factors of pro-environmental behavior from the individual–society path. The purpose is to explore the differences of social capital in influencing individual environmental behavior choices and put forward effective suggestions to promote group pro-environmental behavior. At the same time, previous studies rarely put public domain pro-environmental behavior and private domain pro-environmental behavior in the same framework. Simultaneously analyzing the impact of social capital on two types of pro-environmental behavior is also one of the innovations of this paper. The rest of the paper is arranged as follows: Section 2 is a literature review and research hypothesis, which analyzes the influence mechanism of social trust, social norms, social network, and social participation on people’s pro-environmental behavior from a theoretical perspective, and it puts forward the hypothesis to be tested. In Section 3, we indicate the data sources, set the regression model, and define variables. Section 4 is the analysis of the results, which empirically tests the impact of social capital on public and private domain pro-environmental behavior and further explores the mechanism of action. Section 5 summarizes this paper.

## 2. Literature Review and Research Hypothesis

### 2.1. Definition of Pro-Environmental Behavior

Pro-environmental behavior is also called environmentally friendly behavior or environmental protection behavior. Stern [16] defined it as people’s activities to protect the environment or prevent environmental degradation. Kollmuss and Agyeman [17] defined pro-environmental behavior as the behavior of people to minimize the negative impact of their activities on the ecological environment. On this basis, Rice [18] further divided pro-environmental behaviors into private and public domain environmental behaviors. Private environmental behavior is an individual’s own or family-centered environmental action closely related to daily life, such as garbage classification, resource reuse, etc. Public domain environmental behavior is a kind of environmental behavior with the nature of “collective action”, including participation in environmental organizations or environmental activities, participation in environmental complaints, and acceptance of environmental policies.

### 2.2. Social Capital and Pro-Environmental Behavior

Social capital is a kind of social composition corresponding to human capital and material capital. It is not only the resource embedded in society by individuals or organizations when conducting related activities but also an important factor for capital owners to obtain reputation, trust, and interests. Social capital has the role of “binder”, and the resources it brings also further improve social efficiency. As a social environment factor, social capital is coordinated and organized and plays a key role in influencing public environmental attitudes and improving individual environmental quality improvement tendencies [19,20,21,22]. Referring to the practice of Stephen and Philip [15], this paper explores the impact of social capital on the pro-environmental behavior of the private domain and public domain from four aspects: social trust, social norms, social network, and social participation.

#### 2.2.1. Social Trust and Pro-Environmental Behavior

Social trust is manifested in the tendency of social members to believe in others, reflecting the closeness of social relations between the two sides. In societies with high levels of trust, individual pro-environmental behavior is more likely to affect other members of the public. Social trust can help both sides affirm the correctness and rationality of pro-environmental behavior, eliminate the obstacles of information exchange and cooperation between individuals, improve the individual perception of environmental knowledge and risk, deepen public understanding of pro-environmental behavior, and promote the public to engage in pro-environmental behavior. Petzold and Ratter [23] found that trust and reciprocity norms in social capital have a significant impact on public attitudes and actions in response to climate change. Hua and Dong [24] found that the direct and indirect effects of social trust on various garbage collection behavior paths are extremely obvious. From the perspective of pro-environmental behavior in the public domain and private domain, since social trust is more related to the behavioral intention of both sides, which mainly depends on the private relationship, the impact of social trust on pro-environmental behavior may be more obvious in the private attribute, while behavior in the public domain involves many subjects, environmental awareness is more inclined to the collective, and the impact may not be obvious. Based on this, the following research hypotheses are proposed:

**Hypothesis** **1** **(H1).** *Social trust has a positive impact on pro-environmental behavior.*

#### 2.2.2. Social Norms and Pro-Environmental Behavior

As an informal system, social norms constrain individual moral behavior and promote consensus among subjects on ideas and intentions. Schwartz [25] found that when individual behavior cognition and responsibility cognition are higher, individual norms will be activated, thereby affecting prosocial behavior. On this basis, the value–belief–norm (VBN) theory was proposed; it refers to a belief generated between human beings and the environment that will activate individual social norms from within, deepen their sense of responsibility, and further adopt pro-environmental behavior [26]. Some people believed that the formal system would change personal behavior in the short term, while the guidance of social norms will lead to potential changes in residents’ environmental attitudes and increased willingness to cooperate in the environment [27]. Han and Cheng [28] examined the impact of traditional media and social media on the relationship between norm perception and pro-environmental behavior, finding that social media can activate subjective norm perception and promote pro-environmental behavior. Unlike the above studies, which focus on the impact of individual social norms on pro-environmental behavior, White and Smith [29] found that group norms also had a positive impact on pro-environmental behavior. In summary, social norms drive people to take pro-environmental behavior from the internal moral level. This kind of pro-environmental behavior should be all-around. Whether it is private or public pro-environmental behavior, social norms will have a positive impact on it. Based on this, the following research hypotheses are proposed:

**Hypothesis** **2** **(H2).** *The improvement of social norms has a positive impact on pro-environmental behavior.*

#### 2.2.3. Social Network and Pro-Environmental Behavior

The social network gives individuals on the network nodes the ability to “accept” and “exert” influence, reflecting the closeness of the relationship between individuals and members in the network, and it can be regarded as a platform for group information exchange. The size of the social network owned by individuals determines the number of social resources. A wide range of social networks can promote the improvement of public trust, norms, and social participation, and it can build a social capital system [30,31]. On the one hand, the exchange of information and resources among network members contributes to the spillover and dissemination of environmental knowledge and enhances individual environmental awareness and attitudes. Some scholars have found through empirical research that positive environmental attitudes do have a significant role in promoting pro-environmental behavior [32,33]. For individuals, as the size of social networks expands, the sources of information for pro-environmental behavior also increase, which will promote the development of holistic standards for how individuals conduct environmental behavior. On the other hand, individuals with large-scale social networks are more likely to occupy a dominant position in the process of social interaction, so they are more likely to express their opinions. In addition, due to the scale effect brought by the expansion of social networks, the concept of environmental protection will be spread through speech or action. Based on this, the following research hypotheses are proposed:

**Hypothesis** **3** **(H3).** *The promotion of social networks has a positive impact on pro-environmental behavior.*

#### 2.2.4. Social Participation and Pro-Environmental Behavior

Social participation refers to the process in which individuals participate actively in social events and interact with people who provide emotional and social support to them [34]. As the expansion of the relationship network, social participation deepens the social attributes of individuals. The improvement of social participation can make a person more convincing, enable individuals to penetrate social interaction, strengthen their perception of environmental risks while improving their sense of responsibility, cultivate their subjective consciousness, and further strengthen their pro-environmental behaviors. On the other hand, environmental informal organizations can help improve individual environmental knowledge and enrich individual pro-environmental behavior. Since social participation does not show a strong sense of boundary, with the improvement of individual responsibility and subject consciousness, pro-environmental behavior choices will be spontaneously formed in the face of environmental “crisis”. Social participation can have a significant impact on both public and private domains. Based on this, the following research hypotheses are proposed:

**Hypothesis** **4** **(H4).** *The improvement of social participation has a positive impact on pro-environmental behavior.*

## 3. Materials and Methods

### 3.1. Data Collection

The data in this research come from the Chinese General Social Survey (CGSS), which is the earliest national, comprehensive, and continuous academic survey project in China. It systematically and comprehensively collects data at multiple levels such as communities, families, and individuals to summarize the trend of social change. It is recognized as authoritative data in many fields such as management and economics. CGSS2013 adopts PPS (Probability Proportionate to Size Sampling) to conduct a comprehensive survey on the basic situation, social perception, moral status, and behavioral awareness of 11,438 households in 32 provinces, 83 prefecture-level cities, and 130 districts (counties) in China. Compared with previous data, the environmental module included in CGSS2013 is more representative and comprehensive for environmental behavior research, so it is suitable for the research on the environmental behavior effect of social capital in this paper. After eliminating the missing values and answering the “unknown” and “other” abnormal values for specific questions, this paper finally obtains a total of 8984 valid samples from 28 provinces, of which 50.9% are male residents while 49.1% are female residents; among these, 60.9% are urban residents, and 39.1% are rural residents. The provincial distribution of the samples is shown in Table 1.

### 3.2. Model Setup

To test the impact of social capital on pro-environmental behavior, this paper constructs two-dimensional data at the Residents–Family level. Referring to the model design of Shao et al. [35], the following regression models are set:

(1) $${\mathrm{envi\_behavior}}_{ij}=\alpha_{0}+\alpha_{1}{\mathrm{trust}}_{ij}+\alpha_{2}{\mathrm{norms}}_{ij}+\alpha_{3}{\mathrm{network}}_{ij}+\alpha_{4}{\mathrm{par}}_{ij}+\gamma_{0}X_{ij}+\varphi_{0}Y_{j}+\varepsilon_{0i}.$$

Among them, the dependent variable is ${\mathrm{envi\_behavior}}_{ij}$, which represents the environmental behavior of the ith interviewee of the jth family. The independent variables include ${\mathrm{trust}}_{ij}$, which represents the individual’s social trust status; ${\mathrm{norms}}_{ij}$, which represents the individual’s social norm status; ${\mathrm{network}}_{ij}$, which represents the individual’s social network scale, and ${\mathrm{par}}_{ij}$, which represents the individual’s social participation. $X_{ij}$ and $Y_{j}$ represent a series of micro-individual characteristics control variables and family characteristics control variables respectively, including gender, age, marital status, health level, education level, religious belief, social fairness, area type of residence, family income, and social class. In addition, i is the number of the interviewee, j is the family of the interviewee, and $\varepsilon_{0i}$ is the random error item.

### 3.3. Measures

#### 3.3.1. Dependent Variable

Environmental behavior (envi_behavior): Referring to the definition of pro-environmental behavior in private and public domains [18], 10 problems reflecting individual pro-environmental behavior in CGSS2013 are found. These problems are composed of respondents’ “never”, “occasionally”, and “often” implementing a certain pro-environmental behavior in the past 1 year. The values of the above answers are assigned to 1, 2, and 3 respectively, and the scale of pro-environmental behavior in private and public domains is constructed (see Table 2). The rotation component matrix is obtained by factor analysis. Firstly, the consistency test of the internal problems of the scale is carried out. The correlation coefficient is distributed between 0.449 and 0.658, and the Cronbach’s α coefficient is greater than 0.700, so the scale has good internal consistency. The KMO value was 0.806, while the *p*-value of the Bartlett spherical test was 0.000, indicating that the data could be factored in. Two factors are extracted by factor analysis. Questions 1 to 5 are clustered on factor 2, and the behavior involved is generally implemented in the private domain. Therefore, the private domain pro-environmental behavior variable private is constructed by summing up. Questions 6 to 10 are mainly clustered on factor 1, and it mainly involves the public domain. Based on this, the public domain pro-environmental behavior variable public is constructed. The higher the score means the better the implementation of private or public domain pro-environmental behavior.

#### 3.3.2. Independent Variable

Social capital (soc_capital). Based on Stephen and Philip’s [15] cognitive classification of social capital, social capital is divided into four aspects: social trust, social norms, social network, and social participation. In CGSS2013, the relevant issues were selected to measure the four aspects of social capital (see Table 3). Among them, social network is measured by two questions, and the final score is obtained by summing up.

#### 3.3.3. Control Variable

To minimize the estimation bias caused by missing variables, this paper controls as much as possible some factors that affect social capital and individual pro-environmental behavior and divides them into individual micro characteristic variable $X_{\mathrm{ij}}$ and family characteristic variable $Y_{j}$, which jointly constrain social capital. Among them, the individual characteristic variables mainly include gender (male value is 1; female value is 0), age, marriage (the married value is 1; the unmarried value is 0), health (very unhealthy value is 1; the relative unhealthy value is 2; the general value is 3; the comparative health value is 4; very healthy value is 5), educational level edu (junior high school and below value is 1; the value of high school/vocational school/technical secondary school/technical school is 2; college, undergraduate, and above value is 3), religious belief religion (the value of religion is 1; the value of non-belief religion is 0), the sense of social fairness fair (the value of complete unfairness is 1; the relatively unfair value is 2; neutral value is 3; the value of comparative fairness is 4; the full fair value is 5). The variables of family characteristics include the region type where the respondents live u_r (the non-rural value is 1; rural value is 0), family income status income, class (the self-perception of the social class from low to high is divided into 10 classes, each assigned 1 to 10).

## 4. Results

### 4.1. Descriptive Analysis

Table 4 lists the descriptive statistics of key variables in the regression equation. It can be seen that the mean values of environmental behaviors in the private and public fields are 9.302 and 5.926 respectively, indicating that there is still a large room for improvement in the implementation of pro-environmental behaviors by the public. The standard deviations of the two reach 2.363 and 1.582, respectively, indicating that there are individual differences in residents’ pro-environmental behaviors. For social capital, the standard deviations of social trust, social norms, and social participation are 1.028, 1.046, and 0.498 respectively, which shows that there is little difference in the public’s possession of these three types of social capital, while the standard deviation of social network reaches 3.216, which shows that there are obvious differences in social network relations among different individuals.

### 4.2. Model Results

#### Association of Social Capital and Pro-Environmental Behavior

We treated private and public domain pro-environmental behavior as dependent variables, social trust, social norms, social networks, and social participation as independent variables, and obtained the regression of model (1); the results are shown in columns (5) and (10) in Table 5. It can be seen from regression (1)–(5) that the estimated coefficients of social trust, social norms, and social participation are significantly positive, but the estimated coefficient of social network is not significant, indicating that the higher the degree of public social trust, the more the compliance with social norms and the higher the degree of social participation, the more the implementation of private pro-environmental behavior. In regression (6)–(10), the estimated coefficients of social norms, social networks, and social participation are significantly positive. This indicates that the greater the public’s compliance with social norms, the greater the scale of social networks, the greater the degree of social participation, and the more inclined to adopt more public domain pro-environmental behavior. The above results show that the improvement of social capital will significantly promote pro-environmental behavior.

Comparing the regression results of pro-environmental behaviors in the private and public domain, it can be seen that the impacts of social norms and social participation on pro-environmental behaviors are both public and private. The improvement of the two helps to promote the implementation of pro-environmental behaviors by the public. However, the impacts of social trust and social networks on pro-environmental behaviors are alternatively different. The former has a significant impact on private pro-environmental behaviors, while the latter has a more significant impact on public domain pro-environmental behaviors.

Control variables have different effects on pro-environmental behavior in the private domain and public domain. The estimated coefficient of gender is significantly negative in column (5), while it is significantly positive in column (10). Some scholars found that the elderly males had significantly higher mean values than the females in terms of individual behaviors but had significantly lower values in terms of social behaviors [36]. This paper also indicates that the male is more inclined to implement public domain pro-environmental behavior, while the female is more willing to adopt private domain pro-environmental behavior. The estimated coefficients of age and age^2^ are only significantly indigenous in the regression of the public domain pro-environmental behavior, indicating that with the increase in age, the private domain pro-environmental behavior will not change much, but the public domain pro-environmental behavior will increase with the increase in age. The estimated coefficient of marital status is significantly positive in the regression of private pro-environmental behavior, while significantly negative in the regression of public domain pro-environmental behavior, indicating that married individuals will increase their willingness to adopt private pro-environmental behavior but will reduce their willingness to adopt public domain pro-environmental behavior.

The estimated coefficient of religious belief is only positive in the regression of public domain pro-environmental behavior, indicating that religious belief can promote public domain pro-environmental behavior to a certain extent, but it cannot change private domain pro-environmental behavior. The estimated coefficient of social fairness is only positive in the regression of public domain pro-environmental behavior, indicating that the stronger the individual’s perception of social fairness, the higher the willingness to implement public domain environmental behavior. The estimated coefficients of health, education, residential area type, family income, and social class are all significantly positive in the regressions, indicating that the higher the individual’s education level is, the better the health status is, and the higher the family income is, the more clearly the consequences and benefits of their behavior are evaluated, and the higher the willingness to engage in pro-environmental behavior.

The above results show that social capital has certain internal differences in affecting environmental behavior. On the one hand, the influence of social norms and social participation on pro-environmental behavior is both public and private. On the other hand, the impact of social trust and social network on residents’ pro-environmental behavior has alternative differences. The former has a relatively significant impact on private domain environmental behavior and does not show a strong correlation with public domain environmental behavior. The latter is the opposite. The specific differences are shown in Table 6.

### 4.3. Mechanism Analysis

#### 4.3.1. Association of Social Capital and Environmental Knowledge

As a subjective cognition of environmental conditions and related issues, environmental knowledge is the ecological and environmental endowment of the public. Environmental knowledge reserve and environmental awareness are decisive regarding public pro-environmental behavior [37,38]. When the public implements pro-environmental behaviors, environmental knowledge plays a guiding role. On the one hand, environmental knowledge can spontaneously guide the public to pay attention to environmental issues and actively choose pro-environmental behavior. On the other hand, as a resource, other public pro-environmental behaviors are received and learned by individuals through social networks, when environmental knowledge can play a screening and feedback role in judging and discouraging environmental destruction when imitating other people’s pro-environmental behaviors. Therefore, this study starts from environmental knowledge to explore whether social capital affects environmental knowledge and further promotes pro-environmental behavior. The following regression model is set:

(2) $${\mathrm{knowledge}}_{ij}=\beta_{0}+\beta_{1}{\mathrm{trust}}_{ij}+\beta_{2}{\mathrm{norms}}_{ij}+\beta_{3}{\mathrm{network}}_{ij}+\beta_{4}{\mathrm{par}}_{ij}+\gamma_{1}X_{ij}+\varphi_{1}Y_{j}+\varepsilon_{1i}.$$

Among them, the dependent variable ${\mathrm{knowledge}}_{ij}$ represents the environmental knowledge of the *j*th family individual *i*, and the mastery of public environmental knowledge is measured by 10 problems in the CGSS2013 database (See Table 7). For specific problems, the respondents who judge the correct answer are assigned to 2, and the error is 0. Based on the consideration of the sample size, while ensuring that there is no large deviation in the variable value, the “Do not know” option is assigned to 1. In addition, Cronbach’s α coefficient of the item reached 0.720, with good internal consistency, and the public environmental knowledge level was measured by summation. Statistics showed that 78.11% of respondents were aware of the harm of automobile exhaust to the environment and health, while only 57.39% of respondents knew correctly about the environmental knowledge that phosphorus-containing detergents could cause pollution to water quality. For other environmental knowledge, respondents also have insufficient and incorrect understanding to varying degrees. Thus, there is still much room for improvement in the public’s reserve of environmental knowledge.

It can be seen that in Table 8, the coefficient of trust is significantly positive. The higher the degree of social trust is, the higher the level of public environmental knowledge, indicating that the improvement of social trust helps to improve the level of public environmental knowledge. The higher the social norms and social networks are, the higher the level of public environmental knowledge. The improvement of the three helps to enrich public environmental knowledge.

With the accumulation of individual social capital, both the internal factors such as social trust or social norms and the external condition such as social network will effectively improve the level of individual environmental knowledge. It also explains the positive transmission mechanism of social capital to promote pro-environmental behavior through environmental knowledge.

#### 4.3.2. Robustness Test

Social structural factors such as social capital are different from physical capital or human capital, which are difficult to observe and measure. To avoid insufficient explanatory power caused by incomplete variable measurement, some problems of CGSS2013 are further selected to replace social trust and social network variables, and repeated regression is used to verify the robustness of the regression results. One is to replace social trust variables. In general, “Do you trust strangers in the current society?” is added to form a new measurement standard; the regression results are shown in columns (2) of Table 8. The second is to replace social network variables. With the question “How is your contact with relatives and friends?” replacing the original question to measure social networks, the regression results are shown in columns (3) of Table 8. It can be seen that the regression results are robust, which once again supports the conclusion that social capital promotes environmental knowledge.

#### 4.3.3. Heterogeneity Test

Due to the multi-level differences among different groups in different regions, the measurement of environmental knowledge level may also be heterogeneous; that is, the impact of social capital on environmental knowledge varies among different groups or regions. To provide more constructive policy suggestions to the government, this paper mainly discusses the heterogeneous impact of social capital on individual environmental knowledge from three dimensions: Subjective class status, Gender, and Regional differences.

##### Subjective Class Status

When individuals have different perceptions of their position in the social class structure, the resulting role differences will directly lead to different social members in prestige, wealth, knowledge, and other aspects, and the corresponding social communication opportunities and social cognition will be limited. People with higher class status generally have good quality and can receive more comprehensive education, which shows higher participation in relevant political and social activities. The attention of such groups to environmental problems in the public sphere will also be improved. When individuals subjectively think that they are at the bottom of society for a long time, their spirit and material will be in a relatively poor state, so there may be insufficient cognition for the deterioration of the public environment and the resulting problems. Therefore, according to the explanatory variable class, the sample is divided into two score intervals of 1 to 5 and 6 to 10. The sample of the former is divided into low subjective class status, and the latter is high subjective class status, which is regressed respectively.

It can be seen from Table 9 that in terms of the improvement of environmental knowledge, individuals with high subjective class status are more susceptible to social capital.

##### Gender

The theory of “Gender role socialization” [39] shows that when society measures the success of women and men by certain standards, society and individuals may jointly construct a gender psychological–behavioral mechanism [40], which will make individuals have a certain impact on the tendency of environmental knowledge learning. Women can show more family-oriented private attributes, while men show social-oriented public attributes [41]. As one of the prerequisites for the public to implement pro-environmental behavior, environmental knowledge is affected by private and public paths. On the one hand, people understand environmental knowledge through relatives and friends; on the other hand, public opinion and network media reports can also indirectly enrich the environmental knowledge of the masses. This makes men and women have different choices in raising the level of environmental knowledge, and the samples are divided into two groups according to men and women for regression.

Table 9 shows that the improvement of social trust has a more significant positive effect on women’s environmental knowledge, which is also highly consistent with the characteristics of women’s social roles. Compared with women, men are mainly affected by social networks.

##### Regional Differences

The local culture such as clan identity, fertility culture, folk belief, and geomancy concept has a certain influence and shaping effect on environmental knowledge and pro-environmental behavior. In terms of environment, southern China has concentrated precipitation and mild climate in summer and autumn, while northern China has less precipitation, combined with over-cultivation and overgrazing, resulting in frequent environmental problems in the northern region, which also causes differences in environmental knowledge and behavior between southern and northern residents. Based on traditional ecological norms and pollution conditions in various regions, the formulation and implementation of local government environmental governance systems and policies will have an impact on pro-environmental behavior choices [42]. On the other hand, based on the different cultural differences between the north and south of China, the residents in the south pay more attention to the cultivation and maintenance of neighborhood relations. Compared with the overall social environment, they rely more on their life circle. The northern region pays more attention to the communication circle, matching fame and status, which is determined by the local political and economic attributes.

In this paper, according to the commonly used division ideas in academic circles, the division of the north and south regions is bounded by the 35° north latitude line of the national geographical median line. A total of 15 provinces and cities in Northeast, Northwest, and North China are divided into the northern region, and a total of 16 provinces and cities in East, Central, South, and Southwest China are divided into the southern region (excluding Hong Kong, Macao, and Taiwan). It can be seen from Table 9 that social trust will have a more visible impact on the south, while the north is mainly affected by social networks.

## 5. Conclusions

This paper reveals the potential mechanism among social capital, environmental knowledge, and pro-environmental behaviors. The main contribution of this paper is to construct the influence equation of pro-environmental behavior, including four types of social capital and discuss the implicit relationship between them. At the same time, according to the data, the nature of environmental behavior in private and public fields is effectively divided, and the mediating variable of environmental knowledge is introduced to explore the potential mechanism of a series of pro-environmental behaviors. In addition, we also discussed the heterogeneity of the impact of different social attributes on pro-environmental behaviors to enrich the research conclusions.

The important conclusions of this study can be summarized as follows: (1) The increase in social capital stock does have a positive impact on public pro-environmental behavior. Among them, the positive impact of social norms and participation on pro-environmental behavior is both private and public. Higher levels of social trust have a more significant impact on environmental behavior in the private sector, but they have no significant impact on environmental behavior in the public sector. The improvement of social network mainly has a positive effect on public domain environmental behavior, and private domain environmental behavior is not sensitive to the change of social network. Although all kinds of social capital can affect pro-environmental behavior to some extent, their differences in private and public attributes have not been revealed in previous studies. (2) By means of heterogeneity analysis, we find that the impact of social capital and environmental behavior varies in gender, subjective class status, and region. Due to gender role differences, women have more family and private awareness, so women are more active than men in the implementation of private and public environmental behavior. Second, for individuals with low subjective class status, the impact of social trust and social participation on environmental behavior in the private domain is significantly better than that of individuals with high subjective class status. Finally, compared with the northern region, social capital has a significant impact on pro-environmental behavior in the southern region due to stronger environmental perceptions and better policy support. (3) Environmental knowledge moderates the impact of social capital on environmental behavior. Environmental knowledge reserve plays an important intermediary role in the path of social capital affecting individual pro-environmental behavior. The larger the stock of social capital, the higher the level of environmental knowledge, and the larger the positive effect on the implementation of pro-environmental behavior.

This study has practical value in promoting the formation of individual pro-environmental behaviors. Our research results can help the Chinese government to better incorporate regional institutional background, environmental differences, and individual-level differences in social capital stock into the scope of consideration. Starting from multiple levels of social capital, it will gradually deepen the implementation of residents’ environmental governance behavior, improve the environmental governance system with the public as the main body, and help the long-term and stable development of regional ecological environment from the root.

Firstly, there is the effective use of informal environmental organizations on the dissemination of environmental knowledge, correct guidance, and increased support. Friends of Nature, the Global Village Environmental Education Center and other NGOs strive to present the interdependent relationship between man and nature by building ecological communities and safeguarding legal rights through science popularization, and they make efforts to accumulate public environmental knowledge and form environmental awareness. Due to the limitation of education level and income of the audience, environmental education still focuses on individual groups. In the future, support for informal organizations should be increased and their educational role should be fully utilized.

Secondly, differentiated sustainable development education can activate social capital by focusing on different types of groups of environmental publicity, arousing public environmental emotional resonance, and improving environmental awareness. The study found that there was an inverted U-shaped relationship between age and pro-environmental behavior, which meant that environmental education should pay attention to the differences of different age groups. With the outbreak of the new coronavirus, the radius of individual activities is further reduced compared with the past, which is very unfavorable for the formation of social capital. The use of online curriculum for the sustainable development of young people can weaken the impact of the epidemic on environmental protection education as much as possible, and it can gradually cultivate young people’s environmental subject consciousness. On the other hand, the elderly, as high-risk groups and digital vulnerable groups in the epidemic, need to be concerned. Social alienation and loneliness itself have a strong impact on the elderly. Therefore, based on the planning of the elderly community, the community should strengthen environmental protection education, strive to carry out environmental protection community activities, and enhance the social participation of the elderly. On the one hand, it should improve its social capital, and on the other hand, it should reduce the adverse effects of the epidemic from the psychological level.

In addition, it is important to further improve the mechanism of environmental information communication and stimulate the consciousness of pro-environmental behavior. As an informal way, social interaction has various forms and strong freedom, which highlights the inclusive development of the whole city. The coordination mechanism between the public and the government can also further improve the level of trust in state organs, which is of constructive significance to calm social turmoil under the epidemic. At the same time, it can also raise people’s reasonable understanding of ecological value from irrational to rational, from individual to group level, and help the construction of ecological civilization.

This empirical study provides some meaningful insights for future research, but there are also limitations and ineffectiveness to some extent. Based on the timeliness of data and the differences in individual behaviors over the past 10 years, together with the impact of the COVID-19 pandemic on the global health crisis, the accumulation mode and influence path of various social capital may change accordingly, which is also the field that can be further explored in the follow-up study based on the idea of this study. More factors affecting pro-environmental behavior were included in the influence equation, and more progressive studies were designed combined with major events.

## Figures and Tables

**Table 1:** Sample distribution.

Province	Proportion	Province	Proportion	Province	Proportion
Beijing	5.02%	Anhui	3.93%	Guizhou	2.92%
Tianjin	4.46%	Fujian	2.19%	Yunnan	2.85%
Hebei	2.67%	Jiangxi	3.54%	Shaanxi	2.84%
Shanxi	2.28%	Shandong	5.41%	Gansu	2.17%
Inner Mongolia	1.09%	Henan	5.63%	Qinghai	1.09%
Liaoning	3.86%	Hubei	4.76%	Ningxia	1.06%
Jilin	4.80%	Huna	4.00%		
Heilongjiang	5.42%	Guangdong	1.79%		
Shanghai	5.40%	Guangxi	3.04%		
Jiangsu	4.81%	Chongqing	2.57%		
Zhejiang	4.30%	Sichuan	6.10%		

**Table 2:** Environmental behavior scale.

Category	Activities	Component	
		1	2
Private	1. Waste classification delivery.	0.274	0.543
Private	2. Discuss environmental issues with your relatives and friends.	0.356	0.574
Private	3. Bring your own shopping basket or shopping bag when purchasing daily necessities.	−0.033	0.755
Private	4. Reuse of plastic packaging bags.	−0.110	0.728
Private	5. Actively paying attention to environmental issues and environmental protection information reported in radio, television, and newspapers.	0.426	0.505
Public	6. Donation for environmental protection.	0.608	0.223
Public	7. Actively participate in environmental publicity and education activities organized by governments and institutions.	0.734	0.277
Public	8. Actively participate in environmental activities organized by civil environmental groups.	0.779	0.178
Public	9. Conservation of forests or green spaces at your own expense.	0.606	−0.075
Public	10. Active participation in complaints and appeals for environmental resolution.	0.678	−0.002

**Table 3:** Social capital measurement table.

Social Capital	Activities	Assignment
trust	In general, do you agree that most people can trust in this society?	Very disagree: 1; Relatively disagree: 2; Do not agree: 3; Relatively agree: 4; Very agree: 5.
norms	Assuming that your unit is adjusting wages or working hours, a large number of people, including you, are severely unfairly treated. At this time, if someone wants to ask everyone to talk to the leader and mobilize you together, what will you do?	Strongly support and actively participate: 4; Can participate, but not out: 3; Take a look at the development of the situation and make a decision: 2; Do not participate in any way: 1.
network	What is the frequency of your social activities with your neighbors (e.g., hanging out, watching TV, eating, playing cards, etc.)?	Almost daily: 7; 1 to 2 times a week: 6; Several times a month: 5; About once a month: 4; Several times a year: 3; 1 time a year or less: 2; Never: 1.
	What is the frequency of social activities with other friends (e.g., hanging out, watching TV, eating, playing cards, etc.)?	Almost daily: 7; 1 to 2 times a week: 6; Several times a month: 5; About once a month: 4; Several times a year: 3; 1 time a year or less: 2; Never: 1.
par	Did you vote in the last neighborhood/village election?	Yes: 1; No: 0.

**Table 4:** Sample profile (*N* = 8984).

	Variable	Mean	S.D.	Min.	Max.
Dependent Variable	private	9.302	2.363	5	15
	public	5.926	1.582	5	15
Independent Variable	trust	3.289	1.028	1	5
	norms	2.528	1.046	1	4
	network	8.462	3.216	2	14
	par	0.460	0.498	0	1
Individual Characteristics	gender	0.509	0.500	0	1
	age	48.52	15.83	17	96
	marriage	0.815	0.388	0	1
	health	3.734	1.071	1	5
	edu	1.518	0.758	1	3
	religion	0.104	0.305	0	1
	fair	2.993	1.041	1	5
Family Characteristics	u_r	0.609	0.488	0	1
	income	57,839	128,193	0	10,000,000
	class	4.346	1.655	1	10

**Table 5:** Basic regression and robustness test.

	Private					Public				
	(1)	(2)	(3)	(4)	(5)	(6)	(7)	(8)	(9)	(10)
trust	0.054 **				0.055 **	0.024				0.022
	(2.302)				(2.353)	(1.492)				(1.343)
norms		0.126 ***			0.125 ***		0.072 ***			0.068 ***
		(5.718)			(5.649)		(4.665)			(4.404)
network			0.012		0.008			0.034 ***		0.031 ***
			(1.606)		(1.164)			(6.751)		(6.231)
par				0.172 ***	0.164 ***				0.223 ***	0.209 ***
				(3.587)	(3.427)				(6.667)	(6.253)
Control Variable	Controlled	Controlled	Controlled	Controlled	Controlled	Controlled	Controlled	Controlled	Controlled	Controlled
F	161.5	164.3	161.3	162.3	133.0	83.1	84.9	87.1	87.0	74.2
R^2^	0.178	0.180	0.177	0.178	0.182	0.100	0.102	0.104	0.104	0.110

Note: **, and *** indicate statistical significance at the 1%, and 0.1% levels respectively, in parentheses are standard errors, the same as below.

**Table 6:** Differences in social capital influence.

	Social Trust	Social Norms	Social Network	Social Participation
Private Domain Environmental Behavior	✓	✓	✗	✓
Public Domain Environmental Behavior	✗	✓	✓	✓

Note: “✓” indicates that social capital has a significant effect on private or public domain environmental behavior, “✗” indicates that social capital has no significant effect on private or public domain environmental behavior.

**Table 7:** Environmental knowledge scale.

Knowledge of Environmental Protection	Assignment
Automobile exhaust does not pose a threat to human health.	Correct: 0; Error: 2; Unknown: 1
Excessive use of chemical fertilizers and pesticides can cause environmental damage.	Correct: 2; Error: 0; Unknown: 1
The use of phosphate detergent will not cause water pollution.	Correct: 0; Error: 2; Unknown: 1
Fluoride emissions from fluorine-containing refrigerators can be a factor that destroys the ozone layer.	Correct: 2; Error: 0; Unknown: 1
There is no relation between acid rain and coal burning.	Correct: 0; Error: 2; Unknown: 1
Species are interdependent, and the disappearance of a species produces a chain reaction.	Correct: 2; Error: 0; Unknown: 1
Level 3 air quality means better than level 1 air quality in air quality reports.	Correct: 0; Error: 2; Unknown: 1
Single species of trees are more likely to cause pests and diseases.	Correct: 2; Error: 0; Unknown: 1
In the water pollution report, the water quality of Class V is better than that of Class I.	Correct: 0; Error: 2; Unknown: 1
Increased carbon dioxide in the atmosphere could contribute to climate warming.	Correct: 2; Error: 0; Unknown: 1

**Table 8:** Mechanism analysis (*N* = 8962).

	(1)	(2)	(3)
		Replacement of Social Trust Variables	Replacement of Social Network Variables
trust	0.089 ***	0.080 ***	0.034 *
	(2.873)	(3.787)	(1.746)
norms	0.122 ***	0.073 **	0.091 **
	(4.191)	(2.084)	(1.984)
network	0.020 **	0.045 ***	0.041 ***
	(2.095)	(4.314)	(4.199)
par	0.059	0.023	0.028
	(0.939)	(0.330)	(0.395)
Control Variable	Controlled	Controlled	Controlled
F	153.3	132.64	128.18
Adjusted R^2^	0.204	0.182	0.169

Note: *, **, and *** indicate statistical significance at the 5%, 1%, and 0.1% levels respectively, in parentheses are standard errors, the same as below.

**Table 9:** Heterogeneity test.

	(1)	(2)	(3)	(4)	(5)	(6)
	Knowledge		Knowledge		Knowledge	
	High	Low	Male	Female	North	South
trust	0.227 ***	0.057 *	0.077 *	0.103 **	0.014	0.146 ***
	(3.119)	(1.683)	(1.714)	(2.444)	(0.293)	(3.599)
norms	0.208 ***	0.106 ***	0.145 ***	0.097 **	0.165 ***	0.112 ***
	(2.981)	(3.324)	(3.476)	(2.418)	(3.753)	(2.937)
network	0.034	0.018 *	0.030 **	0.013	0.045 ***	0.000
	(1.443)	(1.742)	(2.174)	(1.032)	(3.175)	(0.030)
par	−0.347 **	0.159 **	−0.001	0.126	0.040	0.072
	(−2.285)	(2.300)	(−0.016)	(1.446)	(0.413)	(0.865)
_cons	12.064 ***	13.554 ***	13.332 ***	13.721 ***	13.426 ***	13.152 ***
	(14.260)	(34.420)	(25.948)	(27.189)	(25.291)	(27.412)
*N*	1731	7231	4562	4400	4284	4678
F	30.69	130.78	70.34	87.93	46.06	119.56
Adjusted R^2^	0.192	0.200	0.174	0.216	0.136	0.274

Note: *, **, and *** indicate statistical significance at the 5%, 1%, and 0.1% levels respectively, in parentheses are standard errors, the same as below.

## Data Availability

Publicly available datasets were analyzed in this study. This data can be found here: http://cgss.ruc.edu.cn/ (accessed on 20 January 2022).
